# Perioperative Systemic Therapies in Resectable Non-Small Cell Lung Cancer: Opportunities and Challenges

**DOI:** 10.3390/jcm15135009

**Published:** 2026-06-27

**Authors:** Natalia Kwiatkowska, Alain Gelibter, Piotr Gabryel, Cezary Piwkowski

**Affiliations:** 1 Department of Thoracic Surgery, Poznan University of Medical Sciences, 60-569 Poznan, Poland; cezary_p@hotmail.com; 2 Doctoral School, Poznan University of Medical Sciences, 60-569 Poznan, Poland; 3 Department of Oncology, Sapienza University of Rome, 00161 Rome, Italy; alain.gelibter@uniroma1.it

**Keywords:** non-small-cell lung cancer, immune checkpoint inhibitors, perioperative IO, progression of the disease, randomized clinical trials, surgical cancelation

## Abstract

**Background/Objectives:** Recent advancements in immunotherapy have significantly reduced recurrence rates and improved distant outcomes of patients with non-small cell lung cancer. This review synthesizes literature from 2020 to 2025, concentrating on preoperative immunotherapy outcomes. **Methods:** We analyzed treatment regimens, focusing on primary endpoints, the percentage of patients who underwent initial surgery, type of surgery, R0 rate, immune-related adverse events and chemotherapy-related toxicities as well as the rate of surgery delays and cancelations. **Results:** Our findings emphasize the importance of optimizing patient selection, effectively managing adverse events, and implementing strategies to minimize surgical delays and cancelations. **Conclusions:** We defined areas for improvement, such as increasing the implementation of minimally invasive surgeries and avoiding pneumonectomies. These priorities are essential for increasing the efficacy of immunotherapy in surgical settings for NSCLC, and improving patient outcomes.

## 1. Introduction

Lung cancer remains the most common cause of cancer deaths worldwide [1,2], with non-small cell lung cancer (NSCLC) accounting for 85% of all lung cancers [1]. Depending on the stage, histologic type, and genetic mutation status, NSCLC can be treated with surgery, and/or with radiation therapy, chemotherapy, immunotherapy, and molecularly targeted therapy [3]. Surgical resection is the primary treatment for low-grade NSCLC (stage I–IIA), but there is a significant group of patients operated on at higher grades (IIB–IV) [3,4]. Perioperative systemic treatment can improve the outcome of patients with locally advanced NSCLC, and until recently, the standard perioperative therapy for stage II and III NSCLC was cisplatin-based chemotherapy [5]. Preliminary studies indicate that the use of immunotherapy [6,7,8,9] in selected groups of patients undergoing surgery can significantly reduce the risk of recurrence and improve distant outcomes by leading to downstaging, so that doubtful cases can be turned into resectable ones [3,10,11]. To date, the US FDA approved neoadjuvant and perioperative immunochemotherapy regimens include nivolumab, pembrolizumab, and durvalumab based on clinical trial results of CheckMate-816, CheckMate-77T, KEYNOTE-671, and AEGEAN. The final analysis of RATIONALE 315 [12] and Neotorch [13] studies demonstrates promising outcomes for perioperative tislelizumab, and toripalimab; however, these drugs do not currently have FDA approval for NSCLC. While preoperative systemic therapy can potentially adversely impact patients’ overall health status and worsen surgical conditions, the results of studies are inconclusive [10]. According to Houda et al., up to 20% of patients will not receive surgery due to treatment-related toxicity, progression of the disease or unresectable tumor [11]. Therefore, this paper aims to examine reasons for surgical challenges and strategies to mitigate them. Our objective is to analyze the existing literature, with an emphasis on phase III randomized clinical trials.

## 2. Materials and Methods

**Figure 1:**
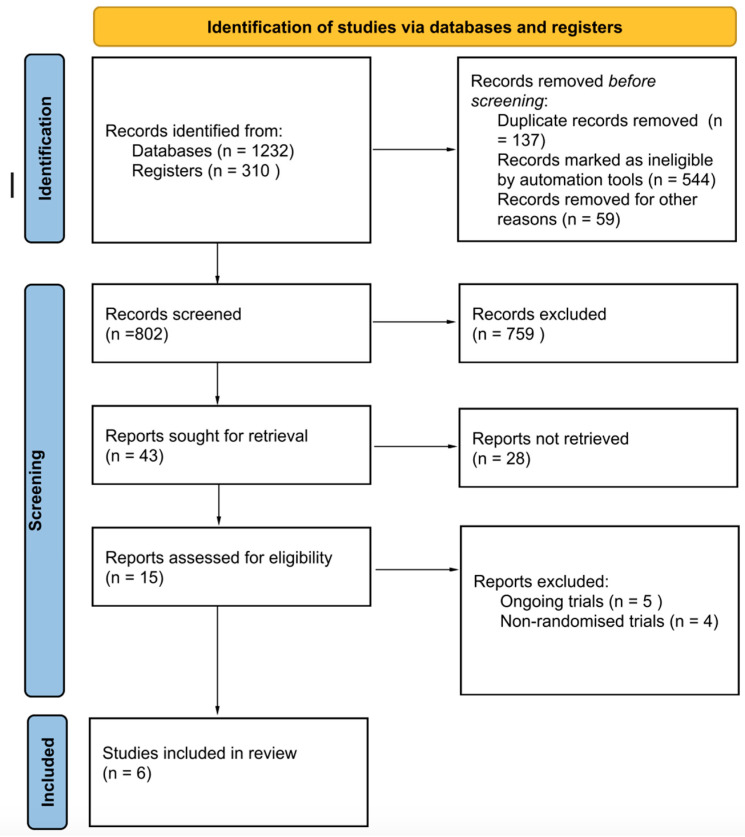
PRISMA 2020 flow diagram for new systematic reviews.

We followed the Preferred Reporting Items for Systematic Reviews and Meta-Analyses (PRISMA) guidelines during the literature review (Figure 1) and reviewed the latest literature on the subject and conducted a review of the existing literature from 2020 to 2025 using PubMed, Embase, and Cochrane Central Register of Controlled Trials. We used the Medical Subject Headings (MeSH) search for: “Non-small-cell lung cancer”; “complications”; “immunology”; “mortality”; “therapy”. The search was restricted to English-language articles, prioritizing the evidence from randomized clinical trials. All titles and abstracts of the identified articles were evaluated to identify those specifically focusing on preoperative immunotherapy. We analyzed treatment regimens, the primary endpoints, percentage of patients who underwent initial surgery, type of surgery, R0 rate, potentially immune-mediated adverse events, and rate of surgery cancelations. The inclusion criteria were phase III RCTs focusing on perioperative immunochemotherapy for NSCLC. We excluded non-randomized studies, abstracts, case reports, ongoing trials without published results or without surgical analysis, as well as clinical trials evaluating the use of radiotherapy, vaccines or those focusing on EGFR and/or ALK mutations only.

## 3. Results

### 3.1. Tumor Staging and Imaging

In the AEGEAN trial, tumor assessment was conducted using Response Evaluation Criteria in Solid Tumors version 1.1 (RECIST v1.1) [8]. In the CheckMate-816 trial tumors were evaluated at baseline using positron emission tomography (PET) and computed tomography (CT). Patients with suspicious mediastinal lymph nodes required pathological confirmation through mediastinoscopy, thoracoscopy, or endobronchial ultrasound (EUS). Additionally, patients with stage II or higher and those with suspected brain metastases had magnetic resonance imaging (MRI) or CT of the brain [6]. In the CheckMate-77T trial, tumor evaluation followed Response Evaluation Criteria in Solid Tumors, version 1.1, using PET and CT with contrast at screening [9]. In the KEYNOTE-671 trial, all participants underwent chest and abdominal CT as a preferred option or MRI as well as brain MRI with contrast and FDG-PET or FDG-PET/CT during sceening [7]. The RATIONALE-315 stage was defined by the Union for International Cancer Control staging system, V8 [10]. In the Neotorch trial baseline tumor imaging included CT or MRI of the chest and of other anatomic sites with suspicious lesions including abdomen and pelvis [13].

### 3.2. Surgery

Surgery following neoadjuvant treatment presents significant challenges, including increased complications due to potential fibrosis and bleeding, as well as difficulties in lymph node dissection [14]. Furthermore, it requires technical precision to achieve complete tumor resection without positive margins. Therefore, it is crucial to choose the optimal surgical approach and carefully plan the type of procedure, focusing on minimally invasive access and avoiding pneumonectomies. All of these factors influence the completeness of resection and the recovery time before the possible adjuvant phase of treatment.

- Surgical access

In the AEGEAN trial thoracotomy and minimally invasive procedures were performed at similar rates, accounting for 40% each in both durvalumab and placebo groups [8]. In the CheckMate-816 trial thoracotomy was performed twice as frequently as minimally invasive surgery in the nivolumab group at 59.1% and 29.5%, respectively, with conversion rates at 11.4% [6]. There were no data regarding surgical access for CheckMate-77T [9], KEYNOTE-671 [7], RATIONALE-315 [10] or Neotorch trials [13].

- Type of procedure

Lobectomy was the most frequent surgical procedure across all the studies, accounting for approximately 65–80% of cases. Pneumonectomy was the second most common procedure. In the AEGEAN trial planned pneumonectomy at admission was an exclusion criterion. Nonetheless, pneumonectomy was performed in 7.1% of patients, following lobectomy, with sleeve lobectomy at 0.5% [8]. In the CheckMate-816 trial pneumonectomy was the second most common procedure for the nivolumab group at 16.8% and in the CheckMate-77T at 9%, with sleeve lobectomy at 1.3% per the CheckMate-816 trial [6,9]. For pembrolizumab, pneumonectomy was performed in 11.4% of patients [7] and for toripalimab, it was 9% equivalent to sleeve resection rates [13]. Compared to the placebo group, pneumonectomy was significantly less frequent with nivolumab [6,9] while rates for durvalumab, pembrolizumab and toripalimab were similar [7,8,13]. In the KEYNOTE-671 and CheckMate-77T trials, segmentectomy rates were reported to range from 0.3% to 1.1% [7,9]. Wedge resection rates, observed in KEYNOTE-671, CheckMate-77T and AEGEAN trials, ranged from 0.3% to 0.6% [7,8,9]. Other procedures included bilobectomy and regional lymph node dissection alone. There were no data regarding surgical access for RATIONALE-315 [10].

- Evaluation of pathological response

For the AEGEAN trial, pathological response was evaluated according to recommendations of the International Association for the Study of Lung Cancer [8,15]. In the CheckMate-816 and CheckMate-77T, Immune-Related Pathologic Response Criteria (irPRC) were applied [6,9,16]. Additionally, in CheckMate-816, it was recommended to sample at least five lymph node stations, including a minimum of three mediastinal nodes. In the KEYNOTE-671 trial, the preferred method for mediastinal lymph nodes staging during surgery was resection of all accessible ipsilateral mediastinal lymph nodes, labeled by level. As a minimum alternative, resection of level seven and one lobar-specific mediastinal lymph node level was acceptable [7]. In the RATIONALE-315 and Neotorch trials, Blinded Independent Pathology Review (BIPR) was recommended for pathological assessment [10,13].

- Completeness of resection

In the AEGEAN trial, among all the patients who underwent surgery in the durvalumab group, 94.7% had complete resection compared to 91.3% in the placebo group. R1 rates were lower for durvalumab, while R2 were equivalent for both groups [8]. In the CheckMate-816 trial, R0 resections were 83.2% for nivolumab compared to 77.8% for placebo, with R1 rates at 10.7% and 15.6%, respectively, and R2 similar at 3.4% and 3% [6]. In CheckMate-77T, R0 rates were 89.3% for nivolumab versus 90.4% for placebo, with R1 higher in nivolumab at 9.6% versus 6.2% for placebo, and R2 rates at 1.1% for nivolumab and 3.4% for placebo [9]. In KEYNOTE-671 among patients who underwent surgery, 92% in pembrolizumab and 84.3% in the placebo group achieved complete resection, while R1 rates were 5.2% and 9.8% and R2 rates at 1.2% and 1.3% [7]. In the Neotorch trial, complete resection was achieved in 95.8% of patients compared to 92.6% in the placebo group [13]. In the RATIONALE-315 trial, 84.1% of patients received definitive surgery compared to 76.2% in the placebo group [12].

### 3.3. Surgical Delay

One of the aspects of efficacy in neoadjuvant immunochemotherapy trials is time to surgery, which may reduce the risk of disease progression and maximize surgical volume. In the AEGEAN trial, a surgical delay is defined as surgery occurring more than 40 days after the last dose of neoadjuvant treatment. The length of delay is calculated as the time beyond the protocol window of 40 days to the date of surgery. Among all the patients in the durvalumab group, 14.5% experienced delayed surgery, primarily due to logistical reasons (7.7%). Adverse events caused delays in 2.7% of patients, and unresolved toxicity from neoadjuvant treatment led to delays in another 2.7%. Regarding the duration of the delays, 7.7% had delays, 3.7% had delays of 2–4 weeks, 1.7% had delays of 4–6 weeks and 1.2% had delays exceeding six weeks [8]. In the CheckMate-816 trial, surgical delay is defined as the time from the last dose of neoadjuvant treatment to surgery exceeding 6 weeks. Among all the patients who underwent surgery in the nivolumab group, 20.8% experienced delayed surgery. The most common reasons were administrative issues (11.4%), followed by adverse events (4%). Among patients with delayed surgery, 54.8% had delays of two weeks or less, 25.8% had delays of 2 to 4 weeks after the per-protocol timeframe, 9.7% had delays of 4 to 6 and 9.7% had delays exceeding 6 weeks [6]. In the CheckMate-77T trial, 15.7% of the operated patients in the nivolumab group had delayed surgery, most commonly due to logistic issues and adverse events (both at 3.5%). Of these, 55.6% of patients had delays of 2 weeks or less, 25% had delays of 2–4 weeks, 5.6% had delays of 4–6 weeks, and 13.9% had delays exceeding 6 weeks [9]. In the KEYNOTE-617 trial, surgery was expected to be performed no later than 20 weeks after the first dose of neoadjuvant pembrolizumab. In the pembrolizumab group, 4.9% of patients experienced delays in surgery in total, compared with 7.6% in the placebo group. The most common reason for delay was adverse events, accounting for 3.1% of delays in the pembrolizumab arm versus 2.5% in the placebo arm. The second most common reason for delay in the pembrolizumab arm was administrative issues, accounting for 1.5% of all delays [17]. In the interim analysis of RATIONALE-315, surgical delay is defined as surgery occurring more than 6 weeks after the last neoadjuvant treatment dose. In total, 5.3% of patients receiving tislelizumab experienced surgical delay due to treatment-related adverse events [12]. For Neotorch, no data regarding surgical delay are available.

### 3.4. Surgical Cancelations

Patients undergoing curative-intent surgery for lung cancer face survival challenges due to both the operation and the underlying disease. The quality of surgery is evaluated by the achievement of complete resection (R0). Potential disadvantages of the neoadjuvant approach include treatment-related toxicity, disease progression, clinical deterioration due to comorbidities or unresectability of tumors [11].

- Immune-related adverse events (irAEs) and chemotherapy-related toxicities

Adverse events were reported as the causes of surgery cancelation across all analyzed studies. These events encompassed immune-related adverse events (irAEs) and chemotherapy-related adverse events. irAEs were reported in all analyzed trials, ranging from 19.8% to 42.1%. Mild irAEs are associated with better response to treatment, while the severe irAEs are the cause of delay or in extreme cases cancelation of the surgery [15]. irAEs include both endocrine-related adverse effects and non-endocrine adverse effects. Adverse effects reported for durvalumab in perioperative settings included: hypothyroidism, dermatitis, pneumonitis, hepatic events, hyperthyroidism, and colitis, with hepatic events being the most frequent among grade 3 or higher adverse effects [8]. For nivolumab, irAEs included adrenal insufficiency, hypophysitis, diabetes mellitus, hypothyroidism/thyroiditis, and hyperthyroidism. In CheckMate-816 adrenal insufficiency and hypophysitis were exclusively 3 or higher [6] while pneumonitis was the most frequent grade 3 or higher adverse effect in the CheckMate-77T trial [9]. For pembrolizumab, irAEs encompassed hypothyroidism, hyperthyroidism, pneumonitis, infusion reactions, severe skin reactions, colitis, thyroiditis, hepatitis, hypophysitis, myasthenic syndrome, myocarditis, and myositis. Notably one case of pneumonitis led to death in the neoadjuvant phase [7]. In RATIONALE-315, the most common irAEs were immune-mediated skin adverse reactions (17.3%), hypothyroidism (14.6%), and immune-mediated pneumonitis (8%). Immune-mediated pneumonitis was also the most common grade 3 or higher irAE, leading to death in one patient [12]. For toripalimab the most common irAEs were: hypothyroidism, hyperthyroidism, pneumonitis and rash. Hepatic function abnormalities occurred in 1.5% of patients, all of which were grade 3 or higher [13].

Chemotherapy-related toxicities encompassed: nausea, anemia, alopecia, neutropenia, decreased neutrophil count, fatigue, constipation. Anemia, neutropenia and decreased neutrophil count were frequently reported across all analyzed studies, with neutropenia being the predominant grade 3 or higher adverse effects.

- Disease progression

In the analyzed phase III studies neoadjuvant chemoimmunotherapy has shown antitumor activity, and superiority over chemotherapy alone in terms of pathological responses and survival [6,7,8,13]. However, a percentage of tumors shows incomplete response to chemoimmunotherapy, linked to early disease progression. Disease progression was the main independent cause of surgery cancelation for durvalumab [8] and nivolumab per CheckMate-77T [9], affecting 6.8% and 5.7% of patients, respectively. In the CheckMate-816 trial, it ranked as the second cause at 6.7% following unfitness for surgery [6]. For pembrolizumab, it was the second cause after adverse events [7]. In an interim analysis of RATIONALE-315 disease progression was reported as the second most common reason for surgical cancelation, accounting for 2.7% of patients [12]. Notably for toripalimab disease progression leading to surgery cancelation was reported only at 2.5%, being outperformed by patient refusal and adverse events [13]. Nonetheless, those rates remained lower than in the chemotherapy group alone across all analyzed studies.

- Clinical deterioration

The primary reason for clinical deterioration resulting in surgery cancelation was inadequate lung function. In patients treated with durvalumab 3.8% were unfit for surgery due to poor lung and/or heart function [8]. For nivolumab, as reported in CheckMate-816 study [6], 1.1% patients were unfit for surgery due to poor lung function. In the case of toripalimab one patient was unfit for surgery for the same reason [13]. For pembrolizumab, 0.3% did not undergo surgery due to worsening clinical status, likely related to disease progression but in the absence of radiographic evidence of disease progression [7]. No data are available regarding clinical deterioration resulting in surgical cancelation for tislelizumab; however, 2.7% of patients did not receive surgery due to treatment-related adverse events in general [12].

- Unresectable tumors

Tumor unresectability is identified as a reason for surgical cancelation in 50% of analyzed studies. For toripalimab, [13] three patients had unresectable tumors at baseline, one patient had unresectable tumor identified during surgery and two other patients in the toripalimab group were at high risk due to tumor enclosing the blood vessels and due to unremarkable shrinkage of tumor. In nivolumab as per CheckMate-816 study, an unresectable tumor was reported in two cases [6]. For pembrolizumab, 1.5% of patients had unresectable tumors, additionally one patient underwent lobectomy; however, metastatic disease was found during intraoperative examination. Therefore the case was classified as unresectable disease [7]. There are no reports of tumor unresectability leading to surgery cancelation for durvalumab and tislelizumab [8,12].

## 4. Discussion

The integration of neoadjuvant and perioperative systemic treatments into clinical practice makes a fundamental shift in the management of patients with resectable NSCLC. It also brings challenges that must be addressed to optimize treatment outcomes. A meta-analysis by Rossi et al. of 11 clinical trials (phase II and III)evaluating immunochemotherapy in NSCLC demonstrated a relative reduction in recurrences for patients treated with chemo-immunotherapy in the neoadjuvant setting compared with those with adjuvant treatment only and suggested that neoadjuvant or perioperative therapy should be a standard of care [18]. Therefore, it is essential to assess the opportunities for improvement in this area across multiple levels of the treatment protocol. Among the analyzed studies, CheckMate-816 was the only trial conducted in a neoadjuvant setting, while the others adopted a perioperative approach.

### 4.1. Qualification for Perioperative Chemoimmunotherapy

Effective patient selection is critical for surgical performance in perioperative immunochemotherapy. Based on findings from CheckMate-816, KEYNOTE-671, and CheckMate-77T trials, the Italian Association of Thoracic Oncology (AIOT) notes greater benefit from immunotherapy in stage III compared to stage II, in N2 involvement compared to N0-N1, in PD-L1 positive compared to negative [19]. These factors might guide the selection of candidates for perioperative chemo-immunotherapy. Poor lung function was the most frequent cause of disqualification from surgery due to clinical deterioration [6,8,13]. Therefore, pulmonary function screening is crucial before enrollment in perioperative treatment.

### 4.2. Surgical Access

Analysis of phase III trials on neoadjuvant immunochemotherapy indicates that the minimally invasive approaches were equivalent to, or outperformed by thoracotomy rates. Nevertheless, minimally invasive approaches were used more frequently in the immunochemotherapy groups than in the placebo groups. In the study assessing ten-year survival outcomes comparing VATS and thoracotomy for major lung resection for stage I–III non-small cell lung cancer, Li et al. concluded that VATS lung resection should be the preferred surgical approach for stage I–III NSCLC due to improved survival outcomes and shorter recovery times [20]. Therefore, a minimally invasive approach should be prioritized in perioperative settings to optimize patients’ recovery and adherence to treatment protocol.

### 4.3. Type of Procedure

Across all the analyzed studies, pneumonectomy was the second most common surgical procedure. According to AIOT, the risk of pneumonectomy should be carefully assessed prior to surgery. The authors suggest that given the risk of pneumonectomy, upfront surgery might be a preferred option to neoadjuvant strategy [19]. Samancilar et al. demonstrated that pneumonectomy is a risk factor for bronchopleural fistula. In their study, bronchopleural fistula rate was 26.5% in the neoadjuvant group with higher incidence after right pneumonectomy, compared with 3.1% in the surgery-alone group [21].

Recent studies suggest that [22] sleeve lobectomy (SL) improves quality of life and extends long-term survival compared to pneumonectomy (PN). A meta-analysis by Li et al. encompassing 27 studies and 14,194 patients demonstrated that sleeve lobectomy is an effective treatment for hilar NSCLC, offering improved long-term survival compared to PN, without increased recurrence rates or postoperative complications. For patients with N0-N1 disease, SL also shows improved survival compared to PN [23]. Therefore, SL should be considered a viable alternative to PN for operable NSCLC. Another surgical option after neoadjuvant immunochemotherapy is anatomical segmentectomy; however, this approach should be reserved only for carefully selected cases, with oncologic principles and R0 resection prioritized [19].

### 4.4. Surgical Delays and Cancelations

Surgical delay is a critical factor affecting outcomes in NSCLC. Samson and colleagues [24] reported that delays in surgery are associated with increased 30-day mortality and decreased median survival compared to timely surgery.

The primary cause for surgical delay across analyzed studies was logistical problems. This factor can be modified and improved through various approaches. In routine clinical practice, outside the controlled trials, these challenges can be even more prevalent. Strategies to minimize delay while medically optimizing higher-risk patients are essential.

Immune-related adverse events (irAEs) were among the causes of surgical delays and cancelations in analyzed trials. The most severe irAEs were pneumonitis and hepatitis. Pneumonitis is a relatively uncommon but potentially fatal complication of immunotherapy, with a median onset of 2.5 months from initiation of treatment [25]. Grade 1 pneumonitis is typically asymptomatic and managed by withholding immunotherapy and initiating oral corticosteroids. Grade 2 pneumonitis presented with new or worsening symptoms, such as shortness of breath, cough, chest pain, and fever, necessitating systemic corticosteroids. Grade 3 pneumonitis affects all lung lobes or more than 50% of the lung parenchyma, and might require additional immunosuppression with infliximab alone or combined with cyclophosphamide. Grade 4 pneumonitis is life-threatening, involving serious respiratory compromise and requiring hospitalization, high-flow oxygen or intubation. In every scenario of pneumonitis as an adverse effect of immunotherapy in NSCLC, exclusion of disease progression and infection is critical [26]. Hepatotoxicity, characterized as elevated liver enzymes, has a median onset of 1.9 weeks in NSCLC. Higher-grade hepatotoxicity is more frequent with nivolumab than pembrolizumab [27]. Grade 1 hepatitis requires weekly monitoring of liver function tests (LFTs). Grade 2 hepatotoxicity require withholding immunotherapy, monitoring LFTs every 3 to 5 days and induction of oral prednisone. Grade 3 and grade 4 hepatotoxicity require withholding immunotherapy, intravenous steroids and gastroenterology consultation. Steroid therapy is indicated until normalization of LFTs and should last from 4 to 6 weeks [25]. Close monitoring of patients treated with immunochemotherapy is essential to identify and manage irAEs. Structured protocols for early detection and treatment are critical for optimizing treatment outcomes. Early disease progression was one of the independent factors for surgery cancelation. Casarrubios et al. found that tumor microenvironment gene expression profiles including elevated IFNG, GZMB, NKG7, and M1 macrophage levels correlate with complete pathological response in resectable NSCLC [28]. These findings suggest potential for a personalized approach in immunotherapy treatment for NSCLC. Further research in this matter is required.

### 4.5. Response to Treatment

The response to neoadjuvant immunotherapy treatment may differ between the primary tumor and metastatic sites such as lymph nodes. Notably, the cases where the primary tumor exhibits poor response to therapy, but lymph nodes demonstrate CPR are challenging. The use of histopathologic features of immune-mediated regression as outlined in Immune-Related Pathologic Response Criteria (irPRC) is recommended for evaluating tumor regression following neoadjuvant immune checkpoint inhibitors [16]. Pathologic response assessment offers the advantage of early indication of therapeutic efficacy within weeks or months rather than years to collect survival data [23]. Standardizing methods for assessing surrogate endpoints, such as major pathological response (MPR), in larger cohorts is critical.

Overall survival (OS) data from KEYNOTE-671 [7], CheckMate-816 [6] and the final analysis of RATIONALE-315 [29] indicate a benefit from adding immune checkpoint inhibition to neoadjuvant chemotherapy. However, the individual benefit of continuing anti-PD1 treatment postoperatively remains unclear due to a lack of long-term data [7,9,20]. The ongoing trials such as ADOPT-lung and PROSPECT-lung aim to address this topic by comparing neoadjuvant vs. perioperative durvalumab and perioperative durvalumab vs. pembrolizumab/nivolumab or atezolizumab [30,31,32].

## 5. Conclusions

The integration of novel neoadjuvant and perioperative systemic treatments represents a significant advancement in resectable NSCLC. It also brings challenges that require attention: optimizing patient selection, managing immune-related adverse events, and implementing strategies to prevent surgical delays and minimize the risk of surgical cancelation are critical priorities. This issue requires scientific discussion to enhance treatment protocols and improve patient outcomes.

## Data Availability

The datasets analyzed during the current study are available from the corresponding author on reasonable request.

## Abbreviations

The following abbreviations are used in this manuscript:

NSCLC	Non-small cell lung cancer
irPRC	Immune-Related Pathologic Response Criteria
RCT	Randomized Controlled Trial
